# A Review of the Water and Energy Sectors and the Use of a Nexus Approach in Abu Dhabi

**DOI:** 10.3390/ijerph13040364

**Published:** 2016-03-25

**Authors:** Parneet Paul, Ameena Kulaib Al Tenaiji, Nuhu Braimah

**Affiliations:** 1 Department of Civil Engineering, School of Natural and Built Environments, Faculty of Science, Engineering and Computing, Kingston University, Penrhyn Road, Kingston upon Thames, Surrey KT1 2EE, UK; 2 Department of Mechanical, Aerospace, and Civil Engineering, College of Engineering, Design and Physical Sciences, Brunel University, Uxbridge, Middlesex UB8 3PH, UK; naakulaib@yahoo.com (A.K.T.); nuhu.braimah@brunel.ac.uk (N.B.)

**Keywords:** water–energy nexus, cogeneration, desalination

## Abstract

Rapid population increase coupled with urbanization and industrialization has resulted in shortages of water in the Middle East. This situation is further exacerbated by global climate change due to greenhouse gas emissions. Recent research advocates that solutions to the global water security and scarcity crisis must involve water–energy nexus approaches. This means adopting policies and strategies that harmonize these inter-related sectors to minimize environmental impact while maximizing human benefit. In the case of Abu Dhabi, when designing and locating oil/gas refineries and associated power generation facilities, previous relevant decisions were based on simple economic and geographical grounds, such as nearness to oil rigs, pipelines, existing industries and port facilities, *etc.* The subsequent design and location of water abstraction and treatment works operated by the waste heat from these refining and/or power generation processes was catered for as an afterthought, meaning that there is now a mismatch between the water and energy supplies and demands. This review study was carried out to show how Abu Dhabi is trying now to integrate its water–energy sectors using a nexus approach so that future water/power infrastructure is designed optimally and operated in harmony, especially in regard to future demand. Based upon this review work, some recommendations are made for designers and policy makers alike to bolster the nexus approach that Abu Dhabi is pursuing.

## 1. Introduction

Rapid global population increase coupled with urbanization and industrialization has resulted in the deterioration of water quality and shortages of fresh water supplies, especially in arid regions such as the Middle East [1], of which the United Arab Emirates (UAE) is a part. This situation is further exacerbated by global and regional climate change due to green house gas emissions [2]. Recent research now advocates that any solution to the global water security and scarcity crisis must involve a water–energy nexus approach [3]. This means adopting policies, strategies and key action plans that harmonize these inter-related sectors to minimize environmental impacts while maximizing human benefit [4,5].

For instance, in the case of Abu Dhabi in UAE, previous combined policy/strategy regarding water–energy linkages largely focused on obvious cogeneration issues [6]. Thus, when an oil/gas refinery’s location was selected, it was based largely on economic and geographical issues, such as nearness to oil rigs, oil/gas pipelines, existing population centers and industries, port facilities, shipping routes, *etc.* Further, associated power generation facilities were also largely dictated by these same factors [7,8].

The siting of water abstraction and treatment works based on technologies such as desalination that utilized the waste heat from these refining and/or power generation processes was largely catered for as an afterthought. This meant the water supply abstraction/generation were not located where they are optimally needed. In addition, the water supply and demand did not match the energy supply and demand, since oil/gas prices fluctuate, and water demand varies seasonal, and thus they are often out-of-sync [9]. In this regard, this review study was carried out to show how Abu Dhabi is now trying to integrate its water–energy sectors using a nexus approach so that future water/power infrastructure is designed optimally and operated in harmony to meet forecasted demand.

## 2. Location and Climatology of the UAE

**Figure 1:**
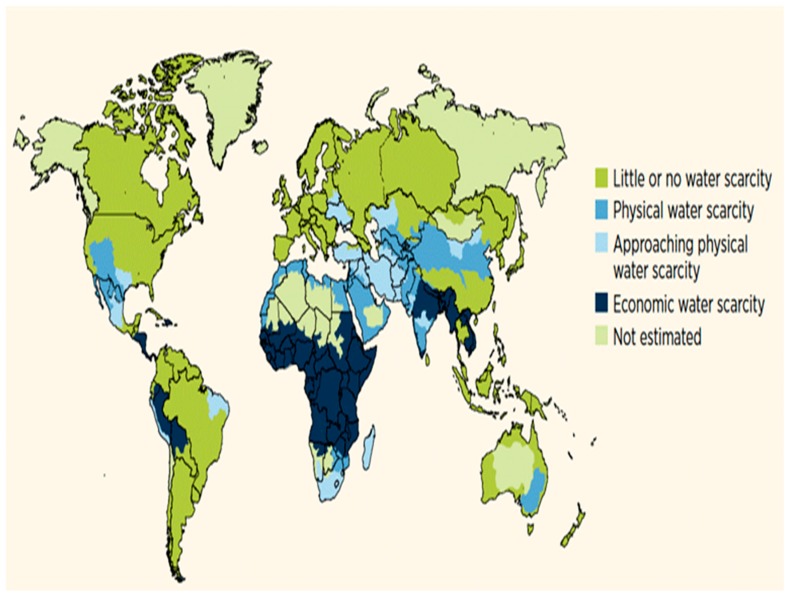
Global water scarcity in physical and economic terms including the Middle East and Saharan North Africa regions (adapted from [11]).

The UAE is located in the hyper-arid climate zone within the Arabian Peninsula that is characterized by low rainfall (*i.e.,* average 100 mm per year) and high temperatures (up to 46 to 50 degree centigrade, especially in summer) and high evaporation rates that can exceed 2000 mm/year. This leads to 75% of precipitation lost to evaporation [10]. Figure 1 shows global water scarcity in physical and economic terms. It is clear that the Middle East and Saharan North Africa regions, which include the UAE, are amongst the most arid zones in the world and thus face the greatest water challenge [11].

**Figure 2:**
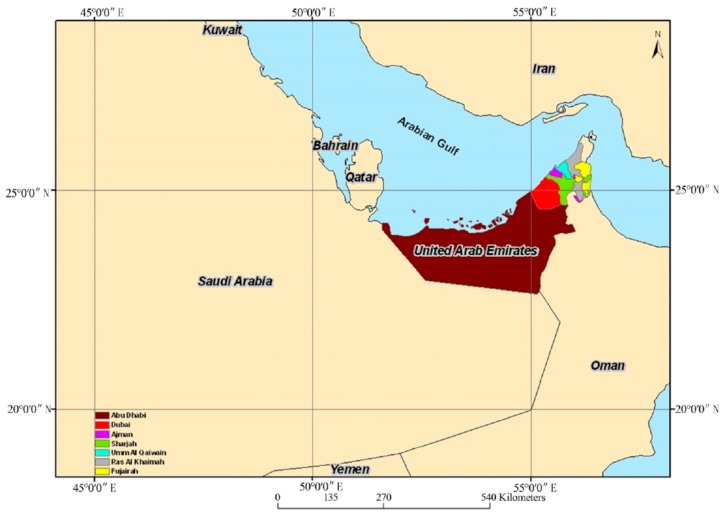
Map of UAE [10].

**Figure 3:**
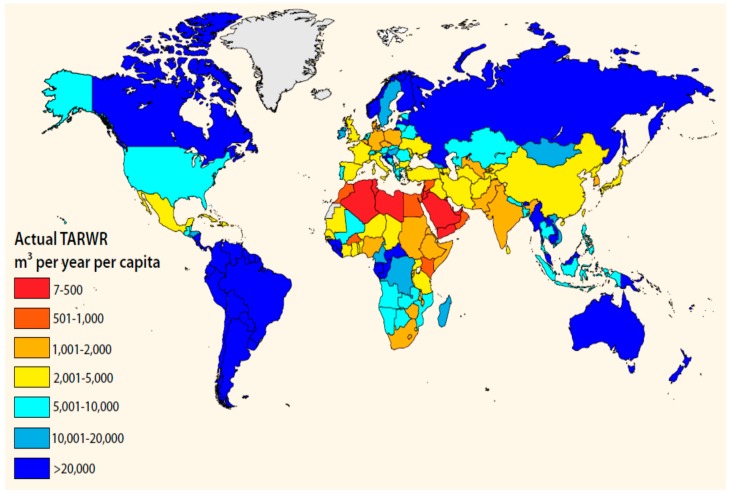
Per capita total annual renewable water resources (TARWR) by country—population data from 2009 [11].

Figure 2 shows a detailed map of the UAE with the seven Emirates boundaries. Even amongst the Emirates that make up the UAE, there is a significant variation in these low rainfall levels, with the highest rate of rainfall occurring in the Northern Emirates such as Fujairah and Ras Al Khaimah since they are near the mountainous recharge areas, while populous coastal and Eastern Emirates like Abu Dhabi and Dubai are receiving the lowest rainfall rate [10]. Due to a low rainfall rate and high evaporation rate, the annual freshwater renewable level per capita in the UAE is very low since the groundwater almost receives no recharge. Figure 3 shows the comparison between the annual per capita renewable water resource levels by country. It is very clear from this figure that the UAE is amongst the very lowest per capita level of countries [11].

The groundwater quantity and quality in Abu Dhabi and Dubai are of the lowest levels when compared with the Northern Emirates, and consequently their water dependencies are now largely based entirely upon desalination solutions as opposed to traditional groundwater sources. Table 1 and Table 2 show the change in this dependency from groundwater abstraction to desalination solutions for years 2000 to 2006 amongst all Emirates (with the Emirates specific local authorities being defined in Section 4) for all water supplies used mainly for non-agricultural purposes. It clearly shows that in the specific case of Abu Dhabi, the rate of dependencies on desalination solutions increased dramatically over this time period from 86% to almost 100%, while its dependencies on groundwater decreased over this same time period from 14% to almost zero. This is due to continuing drops in groundwater levels caused by over abstraction leading to the long term depletion and deterioration of the groundwater. This scenario is occurring in all Emirates but it is at its worst within Abu Dhabi and Dubai since practically now all water supplies come from desalinated only sources [10]. Thus, in the case of the Northern Emirates, the situation has also deteriorated but not to this extent since their rate of dependencies on desalination solutions increased over this time period from 35% to almost 62%, while the dependencies on groundwater decreased over this same time period from 65% to almost 38%.

## 3. Current and Future Industrialization Challenges Facing Abu Dhabi and the UAE

**Figure 4:**
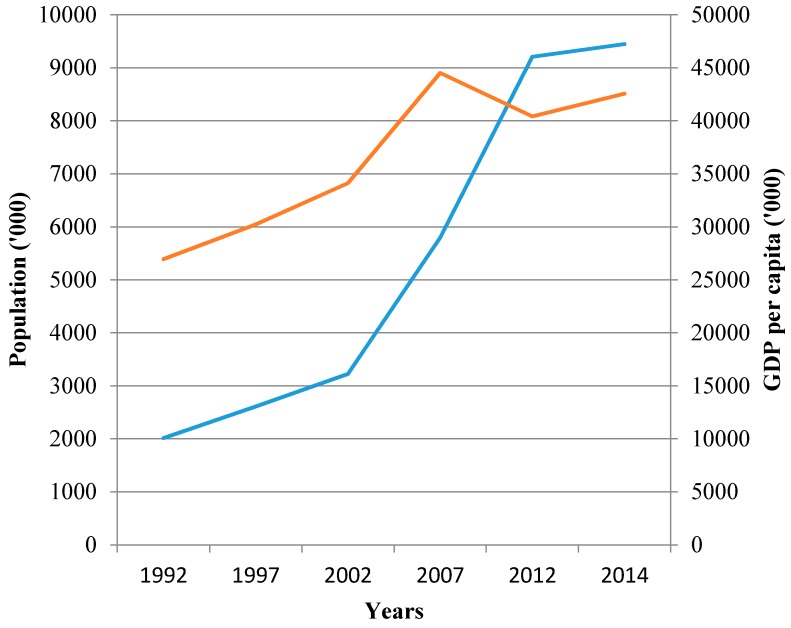
GDP per capita and population growth in UAE [15].

Traditionally, water and power usage in the UAE has always been high due to the arid desert climate that exhibits high temperatures and very high humidity levels. In fact, 65% of the energy usage in the country alone is due to meeting two major demands, namely water provision and the need for cooling by air conditioning [12]. However, since 1971 when the union of the seven Emirates was formally established by his highness the late Sheik Zayed Al Nahyan, the UAE has gone through a unique and sustained economic growth which has led to an abnormal increase in population with subsequent increases in associated water and power needs. In reality, this has meant an exponential population hike from 2.4 million in 1995 to more than seven million in 2011 as recently reported by the Ministry of Foreign Affairs [13] with the bulk of this due to expatriate immigration. Specifically in the last decade, a large part of this economic growth in the country is due to the rapid growth of Dubai as a central hub that focuses on regional trading and financial activities as well as the traditional petro-chemical activities associated with the UAE. In fact, Dubai now accounts for 70% of the non-oil trading activities of the entire UAE [14]. Introduction of the free trade zone concept that has now been adopted by all the main Emirates, namely Abu Dhabi, Dubai, Sharjah and Ras Al Khaimah, has also led to a major increase in the Gross Domestic Product (GDP) in all the different parts of the country. Thus this free trading zone concept has further added to the economic output of the country meaning that there has been a dramatic growth in the jobs market, making the UAE a very attractive destination for many expatriate workers from different countries and across the job spectrum. Figure 4 highlights this rapid GDP growth and associated population growth from 1992 to 2014 [15].

As well as the rapid increase in the population due to economic factors, there has also been a related increase in the living standards of all; an increase in urbanization activities such as construction of residential and commercial properties; an increase in urban industrial activities such as the development of business parks; and an increase in agricultural activities as well as forestry. All of these factors when coupled together have given rise to an increase in the water consumption per capita that now means that water consumption per capita in the UAE is one of the highest in the world and within its own Middle Eastern region [15]. It also means that the UAE has one of the highest carbon emission levels per capita. In fact, the UAE’s carbon emission load has been estimated as twice that of a typical developed country such as the United States, which itself has a figure of 10.5 tons of carbon per capita as an annual average over the last 23 years [14]. In summary, the rapid increase of population due to economic development, and the increase of economic consumption per capita have all led to rapid increases in the demand for water and power alike, which has created a subsequent stress on the country’s water and energy resources, and a stress on the government’s institutional capacity to deliver these required demands.

## 4. Water and Power Sector Organizational Structures for Abu Dhabi and the UAE

As mentioned earlier the UAE consists of seven federal Emirates as depicted in Figure 2. These seven Emirates have slightly different water and electricity organizational structures consisting of local authorities/regulators, government owned companies, private sector stakeholders and semi-privatized entities. The water and power supply chain consists of overarching managing authorities that regulate the producers/providers/suppliers who feed their products down to the transmission and distribution agents. The four major utility authorities are as follows:

- ADWEA: The largest producer of water and power for the Emirate of Abu Dhabi.
- DEWA: The second largest water and power producer with responsibility covering the Emirate of Dubai.
- SEWA: It is responsible for the Emirate of Sharjah.
- FEWA: It is responsible for the Northern Emirates of Ajman, Umm Al Quwain, Fujairah and Ras Al Khaimah.

In terms of power provision, these four utilities have inter-connected power networks known as the Emirates National Grid (ENG). Creation of the ENG has meant power supply is now more reliable and flexible with increased emergency support throughout all of the Emirates. In local terms, the ADWEA is exporting power to the DEWA, the SEWA and the FEWA via signed contracts with agreed annual power quantities supplied by the Abu Dhabi Water and Electricity Company (ADWEC). Furthermore, the UAE’s network is also interconnected to all the Gulf Cooperation Council (GCC) countries through a regional agreement system. The GCC consists of the UAE, Oman, Saudi Arabia, Bahrain and Kuwait. 

### 4.1. Abu Dhabi Water and Electricity Organizational Structure

**Figure 5:**
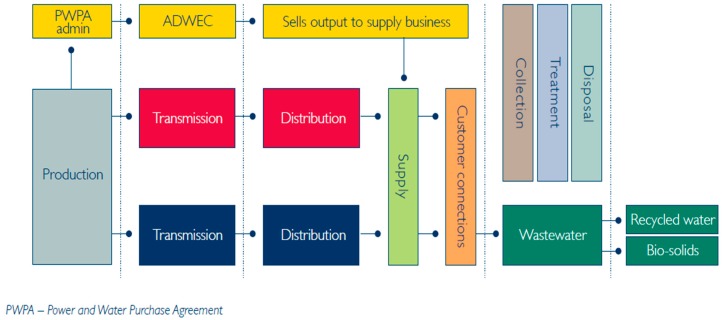
Abu Dhabi water and power sector structure [16].

Figure 5 shows the current power and water sector structure specifically for the Abu Dhabi Emirate [16]. The water and electricity sector in Abu Dhabi was unbundled and restructured in 1999 by the creation of the ADWEA, the ADWEC, the Abu Dhabi Transmission and Dispatch Company, the Abu Dhabi Distribution Company and Al Ain Distribution Company [16]. These companies are 100% government owned entities. During almost the same period, the Independent Water and Power Producers (IWPPs) and Generation and Desalination systems (GDs) were also established as joint venture initiatives between the ADWEA and foreign investors with ownership between the two stakeholder types being 60% and 40% of the partnership respectively [16]. Thus these IWPPs and GDs operate under signed power and water purchase agreements of 20 year’ duration with the ADWEC being the sole buyer of their products [16,17]. The Regulation and Supervision Bureau (RSB) regulates the ADWEC and each of the IWPP’s project company’s. It also issues the operational licenses for these companies and monitors the maintenance of these licenses. 

### 4.2. Renewable/Nuclear Energy in the UAE

The previously mentioned factors that have led to the UAE having a high carbon footprint such as rapid economic growth, population increases, climate change effects, and regional water scarcity impacts, have all meant the UAE government has made a strong commitment to significantly reduce the country’s overall carbon emissions. Thus the UAE government has developed and initiated several practical plans to reduce the UAE’s traditional dependency on fossil fuels, namely oil and natural gas. These plans and initiatives propose the development of renewable energy and nuclear energy as partial replacement energy sources. During a recent Directorate of Energy and Climate Change Event that focused on the UAE’s “Energy Outlook” [14], it was clearly stated that the UAE’s energy profile from 2010 onwards showed a 100% dependency on fossil fuels, which was projected to gradually change in 2020 with the increasing introduction of renewable and nuclear energy. Thus, the anticipated reliance on fossil fuels would be reduced by up to 25% during this period [14].

Despite the high upfront costs of developing renewable energy sources, the UAE has decided to invest heavily in various renewable energy projects not only based in the UAE but also in projects based outside it such as the London Array and Gemasolar projects [14]. The UAE government is also providing direct seed funding in this sector such as under the IRENA renewable projects scheme [14]. 

In terms of Abu Dhabi specifically, in 2008 the Abu Dhabi Council for Economic Development and the Department of Planning and Economy under the Executive Council collectively developed the Abu Dhabi Economic 2030 Vision [18]. The Abu Dhabi Economic 2030 Vision is a long term road map and comprehensive plan for the Abu Dhabi economy that tries to align all policies, strategies and regulations so that the public and private sectors work together to implement this vision’s objective. The main objective of the Abu Dhabi Economy 2030 Vision is to develop a sustainable and diversified economy that has a reduced reliance and shift away from the traditional oil and gas sectors, as well as an increasing focus on the development of alternative knowledge based industries. Accordingly, the government of Abu Dhabi has developed a substitute policy and strategy that aims to diversify the economy while reducing this Emirate’s greenhouse gas emissions. Hence, a number of important initiatives have been established in this regard. Table 3 summarizes the Abu Dhabi based initiatives that are targeted specifically at the water and energy sectors, and that promote green and sustainable development approaches related to water and power industries.

The Abu Dhabi Economic 2030 Vision is in line with the overarching UAE 2021 Vision [19] that was announced in 2010. Both are focused on meeting the following objectives:

- Diversifying of energy sources.
- Securing of energy supplies.
- Ensuring environmental sustainability is embedded in all energy systems.
- Building efficient and financially sustainable energy systems.

As part of the UAE 2021 Vision and national agenda, the UAE has committed that by 2021 it will increase its share of clean energy production by up to 24%, while reducing its carbon emissions by up to 15%, and while trying to achieve full treatment of all wastewater generated by up to 75% [19]. In this regard, Table 4 lists similar green energy commitments made by key countries and blocs such as China, the United States, the European Union and Japan [7]. When comparing these UAE stated commitments with similar ones made by the key countries and blocs listed in Table 4, it is clear that the UAE has made the most comprehensive and specific commitments in this area when compared to the others whose commitments are either less rigorous and so easier to achieve or generally more vague.

## 5. Water and Power Production Capacity in Abu Dhabi

Most of Abu Dhabi’s water and power plants are located near to the coast since traditionally this is where the oil and gas facilities are based whose fossil fuel products are used to power these nearby plants. In terms of the power plant’s themselves they also need large volumes of water to produce the required steam to drive the steam turbines that generate the electricity. The waste heat from the cooled steam, now in the form of hot water, is then normally piped to an adjacent desalination plant that utilizes this energy source to treat seawater in order to produce potable water, some of which is then piped back to be used in the steam generation process. This atypical set-up, which is traditional in the UAE context, is usually referred to as either a “combined cycle” or a “co-generation” arrangement.

The key advantage of this arrangement is that using inter-linked and cyclic processes means major efficiencies are made in the following ways: the fossil fuel does not need to be piped long distances to where it is needed; the waste heat from the steam production used in the electricity generation process is fully utilized to produce clean water for both internal use in the power plant operation and for external supply to main end users; the other aspects of the water treatment works that cannot use the waste heat directly, such as pumps and valve actuators, *etc.*, are powered directly from the adjacent electricity generation plant meaning that further efficiencies are gained; and this arrangement to produce potable water is atypical since the bulk of global water production plants are based on an inefficient single cycle arrangement which usually uses significant amounts of unrecoverable energy.

**Figure 6:**
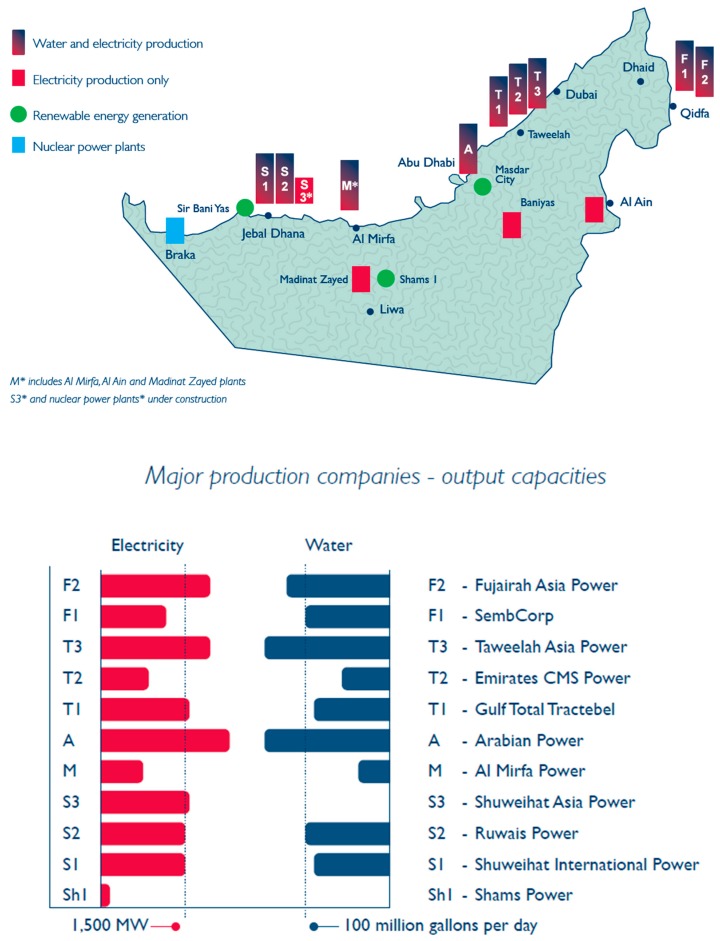
ADWEA power and water generation plants location map and capacity [16].

Figure 6 shows all the water and power generation plants that the ADWEA owns in the Abu Dhabi Emirate, the bulk of which are co-generation based where both water and power are generated in the same plant. The ADWEA also owns and operates further co-generation plant in the Fujairah Emirate located on the eastern coast of the UAE. These particular combined cycle plant produce desalinated water using either a thermal desalination multi-stage flash (MSF) process or a multiple-effect distillation (MED) process. However, the ADWEA also uniquely owns and operates in Fujairah two single cycle desalination plants that use reverse osmosis (RO) technology. These water generation only plant’s, the first of their type in the UAE, have been developed since the locally traditional but globally atypical co-generation system that is commonly adopted and used in UAE was found to be inflexible especially when trying to meet peak loads [16].

Other power only plant that have been developed or are being developed under the Abu Dhabi Economic 2030 Vision are again being used to create greater system flexibility and redundancy. They include several already installed renewable power plant’s using solar and wind technologies that are owned and managed by the Abu Dhabi Future Energy Company (commonly known as Masdar). Further, four nuclear power plants of 1400 megawatts (MW) each are under construction in the western region of Abu Dhabi. Each unit will be separately commissioned in 2017, 2018, 2019 and 2020, respectively, and will be owned and managed by the Emirates Nuclear Energy Cooperation [16].

When looking at the Ministry of Energy’s Annual Statistical Electricity and Water Report for the period of 2011–2013 [20], it is abundantly clear for Abu Dhabi that the actual installed water capacity almost matches the peak capacity meaning that there is no buffer in the system to cater for emergency situations and also little flexibility in water supply scheme. This indicates that the installed capacity will eventually be insufficient to meet peak load demand in Abu Dhabi especially during the summer season when security of supply is essential under severe climatic stress conditions. Conversely in terms of installed power capacity [20], there is a slight over capacity in the system meaning that peak demand can be met. However, since the overall trend of power consumption is markedly upwards, this slight system redundancy will likely be fully utilized in years to come.

### Breakdown of Current Water and Power Consumption and Distribution Profiles

Although in the case of Abu Dhabi almost all the non-agricultural water supplies are now generated from desalinated sources, in terms of total water produced for both agricultural and non-agricultural purposes, the groundwater abstraction level was still high in 2007 and accounted for 64% of total water production [21]. The remaining total water production was made up of desalinated sources accounting for 30% (and used mainly for non-agricultural purposes) and a paltry 6% came from recycled treated sewage effluent sources [21].

The most recent estimates of the breakdown of total water production show that the situation has changed only slightly over the years. Brackish groundwater sources now account for almost 71% of all water produced; with desalinated water accounting for 24%; with 4.8% being treated wastewater, and 0.5% being fresh groundwater [22]. The bulk of this water, amounting to 56% of total production, is still supplied to agriculture. Furthermore, the distribution of water consumption has not historically changed that much either. Agriculture dominates the situation closely followed by domestic usage (*i.e.,* residential), and then followed by forestry usage [21,22].

The distribution of the power consumption is evenly split between residential, commercial and other categories with each representing 30% of the total consumption [20]. The remaining 10% is accounted for by industry. The high consumption rate of power used for residential and commercial purposes is largely due to high cooling loads needed in the building space [20].

## 6. Water and Power Demand Forecasting in UAE—The Regional and Local Context

Extensive research has already been conducted predicting the future water and power needs of the Middle East both at a regional level and in the UAE context. These forecasts are important since they can justify the need for a water/energy nexus approach to these sectors, especially if previous regional and country-specific policies and strategies exacerbated the dichotomies between the water and power sectors rather than harmonizing them. Hence, for instance, a probabilistic based water forecasting approach was used to predict the water demand for Mecca in Saudi Arabia [23]. It made use of historic time series records of water consumption and applied a Monte Carlo simulation to model any associated uncertainties. Mecca probably faces a worse situation then the UAE since it has a highly seasonal visitor count that can dramatically increase the overall population and associated water demands. It also relies exclusively on desalination solutions to meet these needs that are themselves vulnerable to changes in economic activity that itself is greatly dependent on fluctuating crude oil prices [23].

In terms of Abu Dhabi itself, Friedrich *et al*. [24] created an urban electricity usage forecasting model that specifically looked at and isolated the air-conditioning load and its impacts and variables. These variables included the transient thermal response of buildings, the changing climatic conditions and other system perturbations. A short term regression model of electricity consumption was developed specifically for Abu Dhabi using hourly substation level data. It was further combined with data obtained from the Abu Dhabi Urban Planning Council and the National Central Cooling Company to estimate the overall urban cooling load, which is the key driver behind electricity consumption in the UAE. Friedrich *et al*. [24] found that even though only 30% of the annual load was weather dependent, air-conditioning accounted for at least 57% of the annual total electricity load with the summer demand peaking at 75%. In a similar manner, Al-Iriani [25] produced an electricity demand model using time series analysis that focused on air conditioning as a major source of energy consumption especially with variations in climatic conditions. These results can all usefully feed directly into this review study.

Other researchers, such as Assaf and Nour [26], have also tried to create links between the energy and water sectors in Abu Dhabi, although in their work they concentrated on the buildings alone since they are major electricity and water consumers requiring 84.6% and 92.2%, respectively, of the entire demand. They investigated ways of improving the efficiency of Abu Dhabi's built infrastructure usage by employing energy and water performance assessment techniques such as the Estidama pearl rating system. They found that for the selected buildings in their research work, they could potentially produce electricity reductions of 31% to 38% and they could potential water reductions of 22% to 36% based upon their energy and water simulation analysis. Again, these initial findings could feed into this review work.

Other researchers have taken a different approach to this issue, such as Wang *et al*. [27], who looked at the heavy subsidization of utility prices particularly for electricity and water that are specifically used by the Abu Dhabi government as direct ways of supporting industrial growth and lower the cost of living for all. Wang *et al*. [27] estimated potential water and electricity subsidy reductions to lower the overall per capita carbon emissions for this Emirate. This was done using a computable general equilibrium analysis. It was found that electricity subsidy reductions potentially had greater economic and environmental impacts than water subsidy reductions, and it was suggested that these positive economic and environmental gains may incentivize the government to carry out utility subsidy reform in the long term once any negative effects on wages can be offset in other ways [27]. 

### 6.1. Projected Water and Power Demand Profiles

The water and power demand growth rates have been high in Abu Dhabi over the past decade, and they are expected to keep increasing at similar rates until year 2030 at least [28,29]. Based upon the historical water and power usage patterns over this past decade, it is predicted that the forecasted demand growth rate for water will continue to differ from the forecasted demand growth for power. 

Traditionally ADWEC uses forecasting models that estimate the peak demands for water and power in order to ensure the security of supply of both, especially during the summer months when the needs are very high and critical [29]. As depicted in Table 3 and under the government’s sustainable energy strategy, there are a number of standalone power generation plants as government approved projects that are currently under construction to meet forecasted demand such as the solar power plants and the four nuclear power plants mentioned earlier. The first of these four units of 1400 MW will be commissioned in 2017, with progressive units coming on-line in subsequent successive years until 2020. This impending disconnection and decoupling from the traditional way of cogenerating power and water together will mean that the water availability will almost certainly lag behind due to its traditionally strong linkage with power generation. Consequently, construction of standalone water production plant, such as RO desalination units, will also need to be considered if forecasted demands prove accurate.

Historical seasonality issues could make any low demand scenario much worse than anticipated during the summer period. The strong seasonal pattern that emerges from analyzing historical power demand profiles can be largely attributed to the intense need for air conditioning during the summer period because of the high temperature and humidity experienced during this season. The increasing amplitude of seasonal variation between winter and summer demand can be attributed to the increased population growth, standards of living, and industrialization over this 21-year period.

Conversely, historical water demand when analyzed has proved rather constant over the year with much less seasonal variations even though this demand itself has increased in overall terms over the successive years. This difference in the historical power demand profile from its associated water demand profile has always impacted upon the water production system since the water and power production have always been strongly linked by the cogeneration processes. The seasonal divergence of these historical profiles has also been increasing in divergence over the years as each demand increases itself. Thus, it is anticipated that the planned production of large amounts of power-only-plant, such as the 5600 MW from the four nuclear power reactors, will certainly increase the severity of the water production issues, especially during the summer months, unless a suggested concurrent strategy of developing standalone water generation plant is also adopted by the UAE.

The high degree of seasonality in power demand compared to the water demand means that most of UAE’s cogeneration power plant have relatively low utilization of power capacity (or low load factors) when compared with water capacity. This means that there is a dramatic under utilization of overall capacity during the winter season. It must be remembered that cogeneration plants can vary their water output between 70% and 100% of nominal capacity, and their gross power output from between 30% and 100%. In order to have any water production at all, a plant’s power output must be at least 30% of maximum capacity. Consequently, in winter when the peak water demand is as high as approximately 85% of peak summer demand, cogeneration plant cannot switch off entirely as its water capacity is still needed even if its power output is not required to the same extent. During winter, since at least some power production is required to ensure water production, almost all plants must produce at least at their minimum power output, and since electricity demand is very low during this season, nearly all plants tend to output close to this minimum level. This means most power plants have always been under used leading to gross inefficiencies in the ENG system, which if there was sufficient standalone power capacity could allow some power plant to shut down completely during winter while the load was shifted to allow others to operate at peak capacity and efficiency.

### 6.2. Standalone Water Production Plant Based on Desalination Technologies

In order to reduce inefficiencies incurred during the winter months and to increase overall system flexibility and capacity based on forecasted demand scenarios, it is recommended that several standalone water production facilities need to be introduced in a strategic manner by the UAE government. This strategy would then be in tandem with and enhance the flexibility of the standalone power plants as approved projects that are already under construction.

The most suitable option in this regard would probably be to build several water facilities based on RO water production technologies since they are now the most economical desalination systems available in terms of capital and operational costs. Nevertheless, there are many technical considerations and issues to be resolved before any such proposal could be implemented chief amongst them being to get an even better understanding of the link between power and water in the Abu Dhabi context. Other areas needing further work would be optimal desalination technology selection, optimal plant location, and consideration of the whole plant life cycle from inception to obsolescence since these crucial decisions will impact on the local economy, environment, water quality, system flexibility, and power production. The above-mentioned issues that need looking at for specific water plant can be summarized as follows:

- Location selected which will include land availability, specific characteristics of the water intake system, ecosystem, geological conditions, available infrastructure, distance to shipping lanes to avoid risk of pollution, *etc.*
- Location of the point of supply (*i.e.*, required demand), plant overall production capacity and water quality issues.
- Power and steam availability.
- Seawater quality in terms of organic matter, seasonal temperatures, salinity and if located close to sewage outfalls and/or oil and gas exploration or production field/s.
- Environmental impact/s and required mitigation plan.
- Regulatory requirements.
- Issues related to transportation of product water such as distance from the mains distribution network and land elevations.

## 7. Conclusions 

This review study has investigated the water and power issues in Abu Dhabi based upon a nexus approach. The following general and specific conclusions can be made:

- In the last few decades, the UAE has gone through an abnormal growth in population, economy and urbanization that has led to rapid increases in water and power demands. This process will continue for the foreseeable future.
- Fresh water scarcity is very high and there is a lack of renewable water resources development such as wastewater recycling, rainwater harvesting, or aquifer recharge systems.
- The Abu Dhabi government is also trying to reduce its dependency on traditional fossil fuel usage by investing in renewable and nuclear energies as alternatives to meet future power demand. These new sources of energy have themselves both positive and negative aspects that need to be considered in any nexus approach.
- The majority of existing power and water production are based upon cogeneration/combined cycles, whereas this review study advocates that a mix of technologies should be used in order to avoid burning natural gas for water production only when the power demand is low, as it is during the winter season. Hence the highly seasonal power demand profile when compared to its analogous water demand profile leads to system inefficiency and increases in the cost of supply especially for water services.
- There is no doubt that the traditional existing setup of thermal desalination (MSF and/or MED) coupled with power generation increases the overall plant efficiency since cogeneration works by using accessible waste heat to produce potable water as a byproduct from seawater. However, this setup is no longer the optimal one for all power and water plant since during the winter period when power demand is low and subsequently when power generation plant’s need to ramp down, the water demand is still high. This situation creates operational and maintenance constraints for both systems and lowers the power system utilization (*i.e.*, load factor) which leads to lower overall system efficiency and sustainability.
- Previous demand forecast analysis when coupled with historical data analysis clearly show that there is a strong need to decouple power generation and water desalination processes in order to create more flexibility in the system. This is especially true since gas price are high and some power plants can be switched off entirely during winter months.
- The power and water systems cannot be considered separately but rather, must be taken as one integrated system. Therefore any decision regarding the construction of a power plant to meet power demands must also consider its potential impact on water production. Hence, it is recommended that future standalone water plant be developed so that the lag between power demand and system capacity and water demand and system capacity does not grow larger in the future. However any decision to install standalone water plant must also be made ensuring that it does not have any negative impacts on the local and regional environment depending on the abstraction technology employed, *i.e.*, impact on further drops in groundwater levels; allow greater saltwater incursion; promote increases in seawater salinity.
- These proposed standalone water plant would be based on desalination technologies with RO being the favored option at this stage. However selection of the precise desalination technology, as well as plant size, location, *etc.,* would be carefully considered to achieve the optimum power and water needs at the same time, and so that no mismatch in system capacities occurs at any particular location.
- Clearly, there is a need to integrate the water and power demand into a coherent approach that maximizes water coverage while minimizing energy usage. In higher level strategic terms, this suggests the development of a clear process to align these sectors, their projects, and their financial mechanisms with the strategic objectives that serve the overarching country vision (*i.e.*, UAE vision 2021 and Abu Dhabi Vision 2030). In other words, the development of a UAE integrated water and energy short term and long term strategy that ensures the achievement of the individual Emirate objectives/vision as well as the country’s overarching objectives/vision.

Follow-up work from this review should focus on the development of several technical recommendations at the local level geared at helping plant designers and policy makers alike including the production of design flowcharts that clarify these complex water/power inter-linkages in the Abu Dhabi context, especially in terms of new renewable and nuclear power sources that have never been utilized before. Other follow-up research work should concentrate on the creation of optimal demand forecasting methods for water and power designed, adapted and integrated for the specific local conditions and climate of the UAE so that a combined approach is always adopted. This could potentially lead to the real time control of these interconnected systems so that resources are used when needed and diverted to parts of the network or grid where need is greatest.

## Figures and Tables

**Table 1:** Non-agricultural water supplies—groundwater (GW) production rates and change in dependencies in the UAE from 2000 to 2006 (Adapted from data source [10]).

Authority	GW Production in 2000 (MG)	GW Production in 2006 (MG)	Change in Dependencies Rate (%)	GW Produced—Percentage Change in Dependencies	
				2000	2006
**ADWEA**	12,429	476.17	+96.2	14.05	0.27
**DEWA**	2792	3230	−0.16	6.27	4.3
**SEWA**	9907	9407.3	+5.04	47.22	33.22
**FEWA**	10,429.25	6920.46	+33.64	64.98	37.89

ADWEA, Abu Dhabi Water and Electricity Authority; DEWA, Dubai Electricity and Water Authority; SEWA, Sharjah Electricity and Water Authority; FEWA, Federal Electricity and Water Authority.

**Table 2:** Non-agricultural water supplies—desalination production rates and change in dependencies in the UAE from 2000 to 2006 (Adapted from data source [10]).

Authority	Total Production in 2000 (MG)	Total Production in 2006 (MG)	Desalinated Water Produced—Percentage Change in Dependencies	
			2000	2006
**ADWEA**	76,015	176,445.71	85.95	99.73
**DEWA**	41,703	71,703	93.73	95.7
**SEWA**	11,075	18,438.54	52.78	66.22
**FEWA**	5619.77	11,343.5	35.02	62.11
**Total**	134,412.8	27,7942.14		

**Table 3:** The list of initiatives in renewable energy and sustainable development in Abu Dhabi.

Initiative	Location	Year
Abu Dhabi 2030 Vision	Abu Dhabi Emirate	2007
Masdar City	South of Abu Dhabi City	2008
1 MW Wind Turbine	Sir Bani Yas Island, Abu Dhabi Emirate	
10 MW PV Solar Power Panel Plant	Masdar City, Abu Dhabi Emirate	2009
Masdar Institute	Masdar City, Abu Dhabi Emirate	2009
UAE won the bid to host the International Renewable Energy Agency (IRENA)	Masdar City, Abu Dhabi Emirate	2009
Estidama (Green Building Code and Pearl Rating System)	Abu Dhabi Emirate	2010
UAE 2021 Vision		2010
International Water Summit (IWS) launched with the 2013 IWS with the focus on the Water Energy Nexus	Abu Dhabi Emirate	2012
100 MW CSP Solar Power Plant	Western Region, Abu Dhabi Emirate	2013
Desalination Pilots Powered by Renewable Energy	Abu Dhabi Emirate	2014
100 MW PV Plant (approved)	Abu Dhabi Emirate	
28.8 MW Wind Plant (approved)	Abu Dhabi Emirate	
100 MW Water-to-energy Plant (announced)	Abu Dhabi Emirate	

**Table 4:** Green energy: worldwide commitment (data source: [7]).

Country	Green Energy Worldwide Commitment
China	Committed to reduce 16% of energy demand by 2015
US	Adapted new fuel economic standards
European Union	Committed to cut 20% of their energy demand
Japan	Committed to cut 10% of electricity consumption by 2030
